# Psychotraumatology in the Netherlands

**DOI:** 10.3402/ejpt.v4i0.20832

**Published:** 2013-05-02

**Authors:** Eric Vermetten, Miranda Olff

**Affiliations:** 1 Military Mental Health Research Center, Ministery of Defence, Utrecht, The Netherlands; 2 Department of Psychiatry, University Medical Center, Utrecht, The Netherlands; 3 Department of Psychiatry, Academic Medical Center, University of Amsterdam, The Netherlands; 4 Arq Psychotrauma Expert Group, Diemen, The Netherlands

**Keywords:** psychotraumatology, PTSD, Netherlands, history, review

## Abstract

The contribution to psychotrauma literature from Dutch authors has a long tradition. The relatively high lifetime prevalence of trauma and posttraumatic stress disorder (PTSD) is not unique for the Netherlands and does not fully explain the interest in trauma and its consequences. In this overview of psychotraumatology in the Netherlands, we will discuss some of the key events and processes that contribute to the current interest. We outlined the historical basis and development of the field in the Netherlands, including the impact of World War II, the effects of major man-made or natural disasters, engagement in military conflicts, as well as smaller scale traumatic events like sexual abuse and traffic accidents. The liberal and open culture may have reduced stigma to trauma, while other sociocultural aspects may have contributed to increased prevalence. Finally, we describe Dutch psychotraumatology today and how history and culture have shaped the current scientific basis.

The contribution to psychotrauma literature from Dutch authors is relatively large as is their representation at international trauma meetings inside as well as outside Europe (Olff & Vermetten, 2013). The relatively high lifetime prevalence of PTSD of 7.4% and life trauma exposure of 81% (De Vries & Olff, 2009) is not unique for the Netherlands and is comparable to that in the US (Breslau, 2009). Therefore, it is not the prevalence of trauma or trauma related disorders that can justify its interest. Is there a specific sensitivity of the Dutch toward stress and trauma, or is this high interest rooted in history? We can see that the focus on psychotrauma has a long tradition. The contribution of the Netherlands to psychotrauma literature goes back to over 100 years ago. The first Dutch publications on this topic appeared already early in the 19th century and dealt with consequences of transportation accidents or other individual catastrophes, often labeled as traumatic neuroses (see Van der Hart, Hermans, Kleber, & Vermetten, 2012). In this overview, we will discuss some of the key events that contribute to the current interest. We will in particular focus on developments that have contributed to a research domain in psychotraumatology. We will see a returning pattern in which threats are often translated into opportunities for intervention and research.

Being under continuous threat from water, the Dutch learned to reclaim land by building dykes which in itself contributed to a communal resiliency. The Dutch organized themselves into a civil society in which people looked after each other. The country is small, and open in relation to the outside world. The Netherlands is also generally considered to be one of richest and oldest democracies in the world, and is a founding member of the European Union (EU) and North Atlantic Treaty Organization (NATO). The country is known for its modern and liberal policies toward, for instance, drugs, prostitution, homosexuality, and euthanasia and an open mindedness that has been speculated to lead to fewer stigmas attached to mental disorders and traumatic events and thus less reporting bias. At the same time, the degree of urbanization, age distribution as well as ethnic and cultural diversity might be considered as responsible for potentially increasing the true risk for trauma (De Vries & Olff, 2009).

There is a mythical Dutch figure that need mention here, about Hans Brinker, the story of a small boy who stops the sea by putting his finger in the dyke. The dyke is not a metaphor of hope, but rather a symbol of new technology, the story seems a mythical exaggeration of the Dutch resilience.

## The impact of World War II on Dutch psychotraumatology

The interest in psychological trauma in the Netherlands cannot be understood without considering the perspective of the impact of World War II and the German occupation. The impact of World War II can be considered to be this important because the country had remained outside international conflicts since 1815 and had been neutral in World War I.

Hitler publicly broadcasted that he would not invade Holland due to their neutrality during World War I. Yet, the German forces attacked The Netherlands on May 10, 1940, in order to gain access to France to bypass the Maginot Line and entice the Allies to cross the border to attack their spearhead in Belgium. Within 5 days, the Dutch had surrendered. After the surrender, on the same day, a gigantic bombing mission was not recalled and killed 40,000 in Rotterdam. The Dutch government and the Royal family had already left the country and went into exile in London. Dutch citizens endured a harsh occupation. The occupying forces were supported by a minority of the Dutch, at the same time there was active resistance by a small proportion of the population. The Nazi ideological policies led to the deportation of the majority of the country's Jews and other minority groups to concentration camps, with the assistance of the Dutch police and civil service; the Netherlands had one of the highest levels of collaboration with the Nazis during World War II. Of the 140,000 Jews who had lived in the Netherlands prior to 1940, only 30,000 survived the war. One of the riskiest activities was hiding and sheltering refugees and enemies of the Nazi regime, Jewish families like the family of Anne Frank, underground operatives, draft-age Dutch, and others. During the 5 years of the war, in a population of 9 million inhabitants, 210,000 Dutch citizens lost their life as a result of military operations or concentration camps. In addition, there were tens of thousands of deaths caused by malnutrition and the like. The nation was liberated in 1945 largely by Canadian troops, with the assistance of British, Polish, US airborne forces, and French airbornes. In the years after the war, nearly 300,000 Dutch citizens from the former Dutch India came to the Netherlands. They had lived through the Japanese occupation—often interned in camps. World War II has imposed many lasting effects on Dutch society. The Netherlands gave up its neutral state after World War II and became an original member of NATO, the EU, and the UN. On May 4, the Dutch commemorate those who died during the war. Among the living, there are many who still have emotional problems due to the war, in the first generation, but also in the second generation (see below).

The story of Anne Frank has had a big impact on the country. It is amazing how every year thousands of tourists from around the world stand in line, not to the Royal Palace on the Dam, but to visit “Achterhuis”, the place where the Jewish family was hiding. Anne Frank is believed to represent the shame of what has been done to the Jews. This moral consciousness may have played a major role in the attention for psychotrauma.

Until 1940, there was much interest in psychoanalysis (focusing on “inner” trauma). In 1940 the Institute of Medical Psychotherapy was founded in Amsterdam by a group of psychiatrists. They felt a need to anticipate on the consequences of war and occupation in individuals. In this respect, the work of the Dutch psychiatrist Jan Bastiaans needs to be mentioned. He was 23 years old and a medical student when the Germans invaded the country. Bastiaans identified with the problems of the former members of the resistance and regarded himself as an idealistic fighter. He became professor of psychiatry at Leiden University. Trained as a second generation psychoanalyst and a member of a study group in psychosomatic medicine (among whom were Groen, Barendregt and Ladee), he tried to link medical illness with psychological problems and saw trauma coming from outside and imposing a threat to the person. He had been removed from psychoanalytic society. He wrote a voluminous dissertation in 1957 in which he introduced the term “traumatic stress”. The concept of stress was based on Hans Selye's principle of “homeostasis”, taken from stress physiology. Selye's ideas were quite popular in those days and lead to stimulating research (e.g., also see the work of Walter Canon and Rudolf Magnus, to whom the Rudolf Magnus Institute for Neuroscience in Utrecht was later named after).

The work of Bastiaans was performed on people who were approved for a disability pension because of their resistance. He felt that the emotional effects of war experiences could be explained by a failure to return to homeostasis, and saw the delayed consequences as “chronic psycho-somatotraumatic stress” (Bastiaans, 1957). His approach and model was embraced by many that applied for war pensions. For a long time, his work was a classic on the impact of war. Bastiaans was also known for his experiments with psychedelics, such as pentothal, already used by the Americans Grinker and Spiegel (1945) during the war, which he combined with Ritalin and later with lysergic acid diethylamide to allow patients to speak easier and to prevent memory loss. There were dramatic improvements, but the recovery in people with serious difficulties was often temporary. He became quite famous because he was one of the first to recognize the impact of war. His method was controversial and he was increasingly criticized, despite a large crowd of war victims that supported him (Enning, 2009).

By then, the concept of the KZ syndrome (KZ is an abbreviation of Konzentrationslager, referring to concentration camp syndrome) had become very popular. In 1946, the first reports were seen in newspapers and later in scientific journals (Bastiaans, 1974, Cohen, 1972; Musaph, 1973; Van der Hart et al., 2012). They usually emphasized the uniqueness of camp experiences. The concept KZ syndrome would ultimately not prevail, although several characteristics (such as chronic depression and hostility) later returned to the concept of complex PTSD and of enduring personality changes after catastrophic experiences (World Health Organization [WHO], 1992).

## Starting to care for the consequences of the war

For almost 30 years, the psychological impact of the war and the camps had been largely ignored, denied, repressed, and rationalized, enabling outward apparent adjustment. It only became visible and known to the general public after 1975 (Bastiaans, 1974). The post-war reconstruction, the scarcity, emphasis on the resistance together which the Dutch heroism, the tensions between interest groups, and a poorly developed mental healthcare all contributed to this.

A critical breakthrough in recognition of the emotional impact of the war can be considered the heated discussion of the possible release of three German war criminals that thus far had been held in custody in Breda. An important aspect of the public discussion was a documentary by the Dutch filmmaker Louis van Gasteren “Do you understand why I am crying” (Van Gasteren, 1972). The documentary dealt with treatment of one of the survivors of one of the concentration camps and was played the night before the debate about the possible release in Parliament. It was commented on by Bastiaans during a public hearing. Supported with live television, victims of the war united in large numbers against the proposed release of these war criminals.

The increased awareness and openness for the chronic and devastating psychological consequences of the war led to the government's support of specialized psychotrauma care centers and regulation of pensions. In 1973, Stichting Centrum '45 (referring to the year the Netherlands was liberated) started its work in Oegstgeest (derived from the 1971 established Foundation Center Post-concentration Syndrome). From the start, Centrum'45 developed into a nationwide specialist psychological trauma center with attention on the diagnosis and treatment of war victims, refugees, and veterans, now also serving a wider population of trauma victims. The interest in psychotrauma broadened. In 1986, the Institute for Psychotrauma started its work—research, treatment, and after-care—focusing on people confronted with violence and calamities in the work setting (Brom, Kleber, & Defares, 1989; Kleber & Brom, 1986).

In the 1980s, attention was drawn to the phenomenon of *transgenerational traumatization*, i.e., the difficulties of the children of war victims born after the war. In clinical studies, difficulties in the form of depression, difficulty separating from their parents, guilt, etc. were reported (see Aarts, Eland, Kleber, & Weerts, 1991; De Graaf, 1998), but in empirical studies few differences were found between the post-war generation and similar populations (Eland, Velden, Kleber, & Steinmetz, 1990; Schreuder, Ploeg, Tiel-Kadiks, Mook, & Bramsen, 1993; Van IJzendoorn, 2002). This resulted in many heated debates in the media in the 1990s. In 2003, it was again Van Gasteren who had filmed the documentary “The price of survival”, on the impact of the war, in particular on the impact on the post-war generation (Van Gasteren, 2003)..

## Disasters and other major impact trauma in the Netherlands

The Netherlands has had relatively few natural disasters like earthquakes or hurricanes. However, floods have had a major impact especially with the disaster in 1953 when a large part of Zeeland flooded. This collective trauma has led to Delta Works being constructed, a comprehensive set of civil works throughout the Dutch coast. The Netherlands, literally the low countries, may suffer most from global warming with rising sea levels being problematic and weather patterns that may cause the rivers to overflow. The Dutch however are used to this and generally feel proud and confident about their capacity to resist the forces of water.

Later events that contributed to the shaping of Dutch psychotraumatology involved two dramatic train hijackings and hostage-takings in the 1970s, major disasters like the Bijlmermeer aircrash (Gersons & Carlier, 1993), the Enschede fireworks disaster (Meewisse, Olff, Kleber, Kitchiner, & Gersons, 2011; Van der Velden et al., 2006, 2013b), and later the Southeast Asian tsunami in 2004, and airplane crashes (e.g., in Tenerife, 1977; Paramaribo, 1989; Amsterdam Bijlmermeer, 1992; Faro, 1992; Eindhoven, 1996; Waddenzee, 1996; Amsterdam airport, 2009; Tripoli, 2010).

The air disaster shaping psychotraumatology in the Netherlands was the Bijlmer disaster in 1992, when a plane crashed into a residential building in a suburb of Amsterdam, killing 43 individuals in the plane and 43 on the ground, and injuring many more. The disaster was made worse by the fact that the plane exploded and started a large fire after the crash. In 2000, a large cohort study was performed involving 4,806 residents and 2,499 rescue workers. Two phases were identified in the socio-emotional effects of a major disaster: a “honeymoon phase” of public sympathy and solidarity, followed by a “delusion phase”, a backlash in which the non-affected move to the order of the day and the bureaucracy is reinstituted (Gersons, Carlier, & IJzermans, 2000, p. 883). This was also the start of research into debriefing, early interventions and screening (e.g., Sijbrandij, Olff, Reitsma, Carlier, & Gersons, 2006; Sijbrandij et al., 2007) and attention for complex crisis management. Disasters started to be experienced as complex situations, in which crisis management and psychotraumatology were increasingly interwoven. Crises are nowadays given high priority by the government.

Two political murders were of great impact in The Netherlands. In 2002, politician Pim Fortuyn was assassinated during the Dutch national election campaign and in 2004 Theo van Gogh was killed, a Dutch film producer working on a film loosely based on the assassination of Fortuyn. These events affected the political climate dramatically of this safe and tolerant country where ministers were seen cycling to work suddenly turned into a country in which terrorist groups threatened to kill politicians with outspoken non-conformist views and lead to awareness of traumatic stress in generally competent and resilient politicians (Nijdam, Gersons, & Olff, 2010).

## Participation in peacekeeping missions and other deployments

The bitter experience of invasion and occupation during World War II led the Netherlands to become a leading supporter of international cooperation. The Dutch position has been a favorable peacekeeping orientation in support of UN or NATO. Between 1979 and today, over 125,000 Dutch soldiers have been deployed all over the world. It was only after United Nationls Interim Forces Libanon deployment to Lebanon that awareness grew that military deployment also entailed psychological risks. Gradually a system of psychological support arose in the Dutch participation in deployments.

There is little information about posttraumatic stress in soldiers after the police actions in Indonesia and after the Korean War (Bramsen, Klaarenbeek, & Van der Ploeg, 1995; Van Meurs, 1955). The risks of trauma were for some time underestimated, and the prevalence of PTSD is not well known (Engelhard et al., 2007). Although in 1990 there were almost 105,000 troops focused on the defense of Western Europe, there are currently less than 40,000, which is part of a flexible and expeditionary force participating in missions around the world. Since the end of the Cold War, the Netherlands armed forces have been in a continuous process of restructuring and downsizing.

Since the early 1990s, the Department of Defense has facilitated clinical treatment as well as scientific research into the treatment of PTSD. Especially after the “lost innocence” of Srebrenica in 1995, when the impact of the non-use of armed forces became apparent, and due to the severity and chronicity of symptoms that veterans reported from this and previous deployments, it had become clear for society as a whole what the impact of deployments could be. However, it took until 2001 to honor the veterans by the foundation of the Veterans Institute. Post-active military personnel are now allowed to receive psychiatric care. This resulted in a National Network of Civil–Military Cooperation in the Netherlands for care for veterans as well as a new law for veterans (“Veteranenwet”) that solidifies their right for services.

## Trauma outside the public domain

Apart from the major impact events, the most common traumatic events in the Netherlands, as in many other countries, include motor vehicle accidents and the sudden death of a loved one (see for review De Vries & Olff, 2009). Patients presenting at a level one trauma center with motor vehicle accidents victims as the largest category often show considerable levels of posttraumatic stress and other forms of psychopathology (Mouthaan, Sijbrandij, Reitsma, Gersons, & Olff, 2011). At the Academic Medical Center Amsterdam, routine screening for signs of posttraumatic distress has now been implemented in order to refer patients to care (if indicated).

Sexual abuse has slowly gained attention in the Netherlands since the 1980s. In 1988 on behalf of the Ministry of Social Affairs and Employment, research was conducted on 1,000 girls and women who were sexually abused (Draijer, 1988), which was followed by work in the early 1990s about the psychological effects of incest (Albach, 1993; Ensink, 1992). A decade later, a handbook on psychotherapy after sexual abuse (not just incest) demonstrated the growing expertise in this field (Nicolai, 2003) as well as the recognition of the impact of early life trauma. Therapists skilled in the diagnosis and treatment of patients with complex dissociative disorders (e.g., Boon & Van der Hart, 1988) or personality disorders and somatoform disorders (e.g., Van Dijke et al., 2011) found that these disorders generally rooted in chronic (early) childhood trauma. Van der Hart et al. reappraised the pioneering work of Pierre Janet's in the field of complex posttraumatic stress in particular, its dissociation theory and phase-oriented treatment (Van der Hart, 2012; Van der Kolk & Van der Hart, 1989).

One can also see an increased attention for trauma in children, a long neglected research population (Alisic, Schoot, Ginkel, & Kleber, 2008; Bicanic et al., 2012; De Roos et al., 2011; Lanius, Vermetten, & Pain, 2010). Both in research and clinical care, this field has lagged behind in comparison to the study of adults, although already in the 1990s, attention was on the rise (see the work of Wolters, 1991 and Dorrepaal et al., 2012). The recent child abuse in the Catholic Church also surfaced in the Dutch population and led to a large investigation on its impact (Deetman et al., 2011). Globally, there is an interesting initiative by the International Society on Traumatic Stress Studies (ISTSS; istss.org) in collaboration with the European society (ESTSS; estss.org) and other STSSs worldwide to develop guidelines and tools like mobile apps to address the impact of childhood abuse (Koenen, 2013; Schnyder & Olff, 2013).

Around the turn of the 20th century, the awareness was increased due to confrontations with violence in public domain, confrontation of police officers and social workers with high violence. In addition, there were also ongoing developments concerning medical trauma (e.g., Taal, 1999) and trauma caused by war and refugee status (e.g., De Jong, Komproe, & Van Ommeren, 2003; Hondius & van Willigen, 1992). To appraise the development of psychotraumatology in the Netherlands, it also needs be reviewed with reference to this social context. The attention to psychological trauma in the Netherlands as in other countries cannot be separated from changes in the perception of trauma, including the popularization of intrusive images on TV in homes and by what some called the “emancipation of emotions”. The change in attitude of victims, professionals, government and public also played a role. Many victims started to organize themselves, the government became more involved in the acknowledgement of the suffering and managing of the consequences, and the general population, partly due to this emancipation of emotions and the spread of mass media, started to manifest itself (Hermans, 2010; Withuis, 2002).

## Dutch psychotraumatology today

The scientific interest in psychotrauma in the Netherlands as well as health care facilities has increased exponentially during the last three decades. In empirical approaches, the focus has been more on analysis of relevant processes, assessment of problems and difficulties, as well as identification of factors that are most effective in the treatment interventions (for an overview, see Lanius, Frewen, Vermetten, & Yehuda, 2010; Vermetten & Lanius, 2012). Treatment research has a strong emphasis on cognitive behavioral approaches (Gersons & Olff, 2005; Van Minnen, Harned, Zoellner, & Mills, 2012), including eye movement desensitisation and reprocessing (EMDR; Ter Heide, Mooren, Kleijn, De Jongh, & Kleber, 2011). These treatments followed the earlier more psychodynamic approaches that are still more prevalent in some other European countries, e.g., France, Italy, Croatia, and Bulgaria. Brief Eclectic Psychotherapy for PTSD (BEPP) is a good example where most effective elements from psychodynamic approaches are incorporated into a more general cognitive behavioral paradigm (Nijdam, Van der Pol, Dekens, Olff, & Denys, 2013).

There is also an increased interest in neuropsychological and neurobiological research (e.g., Olff, 2012; Olff, Güzelcan, De Vries, Assies, & Gersons, 2006; Olff, Langeland, & Gersons, 2005; Polak et al., 2012a, 2012b; Vermetten & Bremner, 2002) and neurobiology in relation to treatment (Elzinga & Bremner, 2002; Lindauer et al., 2004; Olff et al., 2007; Quidé, Witteveen, El-Hage, Veltman, & Olff, 2012; Vermetten, Vythilingam, Southwick, Charney, & Bremner, 2003; Vermetten, Dorahy & Spiegel, 2007). In the military, after the aforementioned studies on Post Deployment Syndrome and fatigue in Cambodia veterans (De Vries et al., 2000) the Department of Defense also facilitated biological studies in PTSD in alliance with several academic partners to validate the disorder and stimulate research on the disorder. This started with cross-sectional biological studies and later with prospective studies in soldiers, resulting in a flurry of studies that validated what was known and extended the findings because of the use of new designs: Hypothalamus-Pituitary-Adrenal-axis studies (De Kloet et al., 2006) neuroimaging and pain studies (Geuze et al., 2007, 2008), personality (Rademaker, Kleber, Meijer, & Vermetten, 2009), and sleep (Van Liempt et al., 2013). The start of the participation to International Security Assistane Forces deployment was an opportunity to conduct true prospective studies that allowed identification of candidate biomarkers and differentiation of posttraumatic stress, depression, and fatigue (Van Zuiden et al., 2011, 2012), as well as an insight into central brain circuits after deployment (Van Wingen, Geuze, Vermetten, & Fernandez, 2012). These studies have not only contributed to recognition for biological studies but have also demonstrated that a biological basis in PTSD can be identified in Dutch soldiers with deployment related PTSD. Besides the military, the police have also been a fruitful population in the study on the impact of traumatic stress exposure in conjunction with clinical care (Van der Velden et al., 2013a; Lindauer, Olff, Meijel, Carlier, & Gersons, 2006).

The current Dutch psychotrauma research infrastructure has been mapped by Arq (arq.org), the Psychotrauma Expert Center (Gersons, Kleber, & De Pater, 2010) with the aim to discover potential gaps and collaboration on psychotrauma research in the Netherlands and to prepare for national funding of this important area of research. About 20 professors and senior researchers could be identified in the Netherlands that were involved in scientific studies in psychological trauma, often as part of broader research lines (anxiety disorders, experimental psychopathology, emotional stress and personality disorders, epidemiology, and victimology). In addition, approximately 35 research staff were found to be involved in this research domain. In 2010, there were about 65 ongoing research projects and about 60 PhD students involved in research on psychological trauma. A large number of dissertations in the Netherlands have been published on various forms of posttraumatic stress (e.g., see EJPT section PhD summaries). Annually there are approximately 50–60 PubMed-indexed scientific contributions on PTSD from Dutch authors—a rise of over 300% from 10 years earlier.

The report showed two trends. The first trend is that of increasing differentiation. Professionals gradually specialize in a specific area of traumatization. This occurred, for example, in the field of war and combat exposure, child abuse and neglect, sexual abuse, disasters, and mass calamities. The increasing differentiation also means that the field diverges resulting in an apparent lack of cohesion in the field. This also leads to a second trend, i.e., a need for more cooperation and exchange between domains of research and the search for commonality and a commitment to integration of different areas. Already in the 1980s, there was the initiative for a Trauma Study Group (created by Wybrand op den Velde). The need for this was also reflected in the establishment of the Dutch Association for Psychotrauma (Nederlandstalige Vereniging voor Psychotrauma, NtVP; ntvp.nl) and the European Society for Traumatic Stress Studies (ESTSS; estss.org) in which Dutch specialists play a leading role (e.g., Witteveen et al., 2012), and also the creation of Arq Psychotrauma Expert Group (arq.org), where several psychotrauma institutions partner together.

This brief—and by no means complete—overview of history of psychotraumatology in the Netherlands may clarify to some extent the attention given to this topic in this country. With this brief review, we outlined the historical basis and development of the domain of psychotraumatology in the Netherlands. Starting with the notion of being under continuous threat of water, enforcing a communal resilience, we described examples in which several other threats (occupation during the war, disasters, political threats, disasters outside the public domain) have been translated or redefined into opportunities for intervention and stimulated new research. The Dutch have a long-standing tradition of pragmatism, are socially liberal, and are known for their tolerance and openness, potentially reducing stigmatism to trauma. But the Dutch do not stand on themselves. They are well organized in the Netherlands in the Dutch psychotrauma society (NtVP; ntvp.n>), which is embedded in the European traumatic stress society (ESTSS; estss.org), that again is closely affiliated to the international society (ISTSS; istss.org), in all aiming to move the field forward in reducing traumatic stress and its immediate and long-term consequences. In this context, the scientific basis of psychotraumatology can be further expanded and maintained by new young researchers that are inspired by their culture and their educators and are stimulated to conduct new studies.
